# Global Transcriptome Analysis Reveals Distinct Phases of the Endothelial Response to TNF

**DOI:** 10.4049/jimmunol.2300419

**Published:** 2023-11-29

**Authors:** Eike C. Struck, Tatiana Belova, Ping-Han Hsieh, Jacob O. Odeberg, Marieke L. Kuijjer, Philip J. Dusart, Lynn M. Butler

**Affiliations:** * Department of Clinical Medicine, The Arctic University of Norway, Tromsø, Norway; † Centre for Molecular Medicine Norway, Nordic EMBL Partnership, University of Oslo, Oslo, Norway; ‡ Science for Life Laboratory, Department of Protein Science, Royal Institute of Technology, Stockholm, Sweden; § The University Hospital of North Norway, Tromsø, Norway; ¶ Coagulation Unit, Department of Hematology, Karolinska University Hospital, Stockholm, Sweden; ‖ Department of Pathology, Leiden University Medical Center, Leiden, the Netherlands; # Leiden Center for Computational Oncology, Leiden University Medical Center, Leiden, the Netherlands; ** Clinical Chemistry and Blood Coagulation Research, Department of Molecular Medicine and Surgery, Karolinska Institute, Stockholm, Sweden; †† Clinical Chemistry, Karolinska University Laboratory, Karolinska University Hospital, Stockholm, Sweden

## Abstract

The vascular endothelium acts as a dynamic interface between blood and tissue. TNF-α, a major regulator of inflammation, induces endothelial cell (EC) transcriptional changes, the overall response dynamics of which have not been fully elucidated. In the present study, we conducted an extended time-course analysis of the human EC response to TNF, from 30 min to 72 h. We identified regulated genes and used weighted gene network correlation analysis to decipher coexpression profiles, uncovering two distinct temporal phases: an acute response (between 1 and 4 h) and a later phase (between 12 and 24 h). Sex-based subset analysis revealed that the response was comparable between female and male cells. Several previously uncharacterized genes were strongly regulated during the acute phase, whereas the majority in the later phase were IFN-stimulated genes. A lack of IFN transcription indicated that this IFN-stimulated gene expression was independent of de novo IFN production. We also observed two groups of genes whose transcription was inhibited by TNF: those that resolved toward baseline levels and those that did not. Our study provides insights into the global dynamics of the EC transcriptional response to TNF, highlighting distinct gene expression patterns during the acute and later phases. Data for all coding and noncoding genes is provided on the Web site (http://www.endothelial-response.org/). These findings may be useful in understanding the role of ECs in inflammation and in developing TNF signaling–targeted therapies.

## Key Points

- This study investigates TNF-induced endothelial transcriptional changes over 72 h.
- Global analysis revealed discrete acute and delayed temporal gene expression phases.
- IFN-stimulated gene expression was independent of IFN production.

## Introduction

The vascular endothelium is a dynamic interface between blood and tissue that has a role in the regulation of coagulation, blood pressure, solute movement, and inflammation. The resting endothelium is an anti-inflammatory and antithrombotic surface that is unreceptive to interactions with circulating blood cells (1, 2). The cytokine TNF-α is a key driver of acute and chronic inflammation (3, 4) and can bind to endothelial cells (ECs) via TNF receptors 1 and 2. This interaction induces signaling cascades that regulate the activity of several transcription factors, including NF-κB and activator proteins 1 and 2 (5, 6), leading to various cellular responses, such as the expression of adhesion molecules and chemokines that facilitate leukocyte recruitment into tissue (7). TNF can also induce the EC expression of IFN regulatory factors and IFN-stimulated genes (ISGs) (8, 9). Indeed, the concept that ECs are multifaceted conditional innate immune cells has gained traction in recent years (10, 11). However, the global dynamics of the EC response to TNF is not well understood, with existing studies tending to focus on specific gene(s) or phenotypic characteristics [e.g., (12–14)] or global transcriptional changes at a single or a small number of time points (15, 16). The same is true of studies of the TNF response in other cell types, such as the immortalized embryonic kidney cell line HEK293 (17, 18) or fibroblasts (19, 20). Existing studies also tend to neglect the influence of chromosomal sex on cell behavior (21), despite reported differences between male and female ECs, such as in preeclampsia (22) and following exposure to shear stress (23), X-ray–induced damage (24), and hyperoxia (25).

In the present study, we profiled the EC transcriptional response to TNF over an extended time, including 11 time points ranging from 30 min to 72 h after stimulation. We identified TNF upregulated or downregulated genes and used weighted gene network correlation analysis (WGNCA) to decipher coexpression profiles, revealing two distinct temporal phases: an acute response initiated between 1 and 4 h and a subsequent later one between 12 and 24 h. Sex-based analysis revealed a high similarity in response profile between female and male ECs. Several completely uncharacterized genes were strongly regulated during the acute phase of the response, whereas the majority of those in the later phase were ISGs. Our data indicated that this ISG expression was independent of de novo IFN production and subsequent autocrine signaling. All data are available at http://www.endothelial-response.org/, which users can view on a gene-centric or regulation profile basis.

## Materials and Methods

### Materials, data, and code availability

This study did not generate new, unique reagents. The data generated by this study are publicly available. Any additional information required to reanalyze the data reported in this article and resources and reagents are available from the corresponding author upon request.

### Experimental model and subject details

#### Isolation, culture, and sex determination of HUVECs

HUVECs were isolated from human umbilical cords, collected from Karolinska University Hospital, Stockholm, Sweden, as described (26). Ethical approval was granted by *Regionala etikprövningsnämnden i Stockholm* (2015/1294-31/2). ECs were cultured in Medium M199, supplemented with 10% FBS, 10 ml/L penicillin-streptomycin, 2.5 mg/L amphotericin B (all Thermo Fisher, Life Technologies), 1 mg/L hydrocortisone, and 1 µg/L human epidermal growth factor (both Merck). To determine EC sex, transcripts encoding *Ubiquitously Transcribed Tetratricopeptide Repeat Containing, Y-Linked* (*UTY*), were measured by quantitative PCR (qPCR). Cell lysis and cDNA generation were performed using the Two-Step Fast Cells-to-CT kit (Invitrogen, Thermo Fisher) according to protocols. qPCR was performed using TaqMan Fast Universal PCR Mix and target (UTY) primer conjugated to FAM probe (Hs01076483, Thermo Fisher) with 18s rRNA primer (4319413E conjugated to VIC probe, Thermo Fisher) as an endogenous control. qPCR was performed using a Real-Time PCR LightCycler 96 system (Roche Life Sciences). ECs positive for *UTY* expression were classified as male, and those negative were classified as female. Five or six sex-matched biological replicates were pooled to create each sample set (six sample sets, 33 donors in total). Sample sets were A, B, and C (annotated male) and D, E, and F (annotated female). Following sequencing, a low level of Y-linked transcripts was detected in sample set F, indicating an incorrect annotation of one of the constituent donors as female. Thus, this sample was excluded from any subsequent sex-based analysis but was included in non–sex-split analyses.

#### EC treatment, RNA isolation, and sequencing

Pooled HUVEC sample sets were grown to confluence before treatment with or without recombinant TNF-α (10 ng/ml) (Thermo Fisher) in cell culture medium. HUVECs treated with a concentration of 10 ng/ml TNF express key markers of inflammation, such as *SELE*, *VCAM1*, and *ICAM1* (27, 28) and can support leukocyte recruitment (29) while maintaining viability over a prolonged culture period (30). HUVECs maintained morphological features and confluence over the time course. Following the initial treatment with or without TNF, the cell culture medium was not changed for the remainder of the time course to minimize cell exposure to external shear forces and/or temperature fluctuations, factors associated with changes in gene expression in ECs (31, 32). ECs were lysed at 0.5, 1, 2, 3, 4, 6, 8, 12, 24, 36, 48, or 72 h after stimulation, using RLT lysis buffer from the RNeasy Mini kit (Qiagen). All sample sets were treated and processed concurrently. RNA isolation and purification were performed using the RNeasy Mini kit (Qiagen). RNA concentration was measured using a NanoDrop 2000 spectrophotometer, and RNA integrity number was determined using an Agilent 2100 bioanalyzer (RNA integrity number >9 required for inclusion). Library preparation and RNA sequencing were performed by the National Genomics Infrastructure Sweden using the Illumina stranded TruSeq poly-A selection kit and an Illumina NovaSeq 6000S (four lanes, 2× 150-bp reads, including 2Xp kits). The data were processed using demultiplexing. Data storage and initial analyses were performed using server-sided computation supplied by the Swedish National Infrastructure for Computing. Genome assembly used for sequence alignment was as follows: Homo_sapiens.GRCh38.dna.primary_assembly.fa and annotation performed using: Homo_sapiens.GRCh38.96.gtf. Sequence alignment was carried out using STAR/2.5.3a. Gene mapping has been carried out using subread/1.5.2 and the module feature counts. Transcript mapping was carried out using Salmon/0.9.1.

### Quantification and statistical analysis

#### Data normalization and differential gene expression analysis

We used the “DESeq2” package in R to normalize raw gene expression counts (33), which were log_2_ transformed and averaged across biological replicates. Genes with total DESeq count <10 across all samples were excluded, and differentially expressed genes (DEGs) between untreated control and TNF-treated ECs at each time point were identified using the DESeq2 time-series design. In this case, the full model is represented by ∼treatment + time + treatment:time and the reduced model by ∼treatment + time. To correct for multiple sampling for DEGs, *p* values were adjusted using the Benjamini-Hochberg correction. DEGs were defined as those with an adjusted *p* value <0.05 and an absolute fold change (FC) between untreated control and TNF-treated ECs of log_2_ >1. Female and male samples were analyzed separately, thus excluding a possible influence of sex-based discordant baseline expression levels, and data were later merged where stated. In total, comparisons for 11 time points (0.5, 1, 2, 4, 6, 8, 12, 24, 36, 48, and 72 h) were performed. Classifications as DEGs defined by DESeq2 (without application of additional criteria as described below) and associated statistical information are provided in Supplemental Table II.

To see the clustering of samples, multidimensional scaling (MDS) plots were generated to visualize the clustering patterns among samples. Pairwise Euclidean distances between samples were calculated and scaled using classical MDS transformation. The resulting MDS coordinates were used to create a two-dimensional scatterplot using the ggplot package.

#### Thresholding and classification of genes as TNF regulated

To identify genes that were regulated by TNF, we applied several additional criteria. Raw gene counts were normalized to transcripts per kilobase per 1 million reads mapped (TPM), and genes with low expression (maximum TPM <1 at any time point or condition) were excluded. Of those remaining, 4262 genes were classified as DEGs by DEseq2 (Supplemental Fig. 1Bi). When regulated at multiple time points, DEGs had a consistent effect direction (i.e., showing only positive or negative regulation in response to TNF). Genes that were not differentially expressed at two or more sequential time points were excluded from further categorization (Supplemental Fig. 2Bii). Genes were classified as TNF upregulated when the following additional criteria were fulfilled (Supplemental Fig. 2Ci): (1) a low variation of expression over time in untreated ECs (coefficient of variation [CV] of mean expression across time points <0.3), (2) high variation of expression over time in TNF-treated samples (CV >0.3), and (3) a high minimal FC (log_2_ FC >0.7) versus the initial time point (0.5 h). Positive DEGs that did not fulfill these criteria were excluded (Supplemental Fig. 1Cii) because the differential expression between untreated and TNF-stimulated ECs was driven by expression changes in untreated ECs over time (Supplemental Fig. 1Cii). Genes were classified as TNF downregulated when the following criteria were fulfilled: (1) a low variation of expression over time in untreated ECs (CV <0.3), (2) a high variation of expression over time in the TNF-treated samples (CV >0.3), and (3) a minimum log_2_ absolute FC >0.7 in TNF-treated ECs versus control untreated ECs (Supplemental Fig. 1Di). Negative DEGs that did not fulfill these criteria were excluded (Supplemental Fig. 1Dii, 1Diii), because the differential expression was driven primarily by either a TNF-induced delay (Supplemental Fig. 1Dii) or inhibition (Supplemental Fig. 2iii) of expression in control untreated ECs over time.

We applied these criteria to highlight genes with clear, distinct TNF-regulated expression profiles; our classifications should thus be viewed as an illustrative guide rather than as a comprehensive categorization. Indeed, individual regulation profiles for genes of interest are best considered on a gene-by-gene basis.

#### Gene coexpression network construction

A gene coexpression network was constructed using the WGNCA package in R (34). After filtering of low-expressed genes (total DESeq2 counts over the time course <10) and genes with low variation across all samples (CV >0.2), 12,428 genes remained for further analysis. The appropriate soft-thresholding power was selected by applying the “pickSoftThreshold” function with parameter “networkType” set to signed hybrid. Then the correlation network adjacency matrix was calculated using selected soft thresholding of 12 and with parameter “networkType” set to signed hybrid. The adjacency matrix was turned into a topological overlap matrix (TOM), and the corresponding dissimilarity topological overlap matrix was calculated. Finally, average linkage hierarchical clustering was applied according to the dissimilarity topological overlap matrix value, and gene modules were identified using a dynamic tree cut algorithm with the minimum module size of 30 genes. Module eigengenes representing the first principal component of each expression module were calculated for each module. Genes with correlation to the eigengene >0.80 (*p* < 0.05) were featured in the figures and associated subsequent analysis. Heatmaps show log_2_-transformed scaled expression, as calculated by DSeq2.

#### Module visualization

The profiles of each module were visualized using a heatmap of scaled gene expression profiles. Additionally, plots displaying the expression of each gene within a module and the expression of its eigengene were produced (34), which returns the gene in each module with the highest connectivity.

#### Functional enrichment of coexpression modules and DEGs

The Gene Ontology (GO) Consortium (35) and PANTHER classification resource (36) were used to identify overrepresented terms in gene lists using the GO databases (release date July 5, 2023). Plots of GO terms were created using the R package clusterProfiler (37).

#### Additional statistical analyses and Web site development

Graphs were created using the packages ggplot2 (38), base R (39), and GraphPad Prism. Temporal graphs for Figs. 3D, 3E, and 4 and Supplemental Figs. 2 and 3 were created using our Web site tool. Circle plots were created by using the R package circlize (40), and PubMed lookups were performed using the R package easyPubMed by Damiano Fantini (41) (date of lookup January 3, 2023). Figures were assembled using Affinity Designer and Adobe Illustrator.

The following additional R packages were used for mapping, normalization, clustering, and display of the data: readR, dplyr, data.table, matrixStats, NormExpression, edgeR, viridis, RcolorBrewer.

Further statistical analyses and Web site implementation were performed in RStudio (R version 4.0.3) using shinyapps. The following packages were used: shiny (42), shinyjs, gplot, ggplot2, DT, plotly, and ggthemes.

The Web site is available here: http://www.endothelial-response.org/.

#### Web site and data availability resource

Average gene TPM and DEG values (available as log_2_, log_10_, or decimal) for each time point are available in Supplemental Tables I and II, respectively. Raw gene counts are available from https://github.com/PhilipDusart/TNF_timecourse or can be requested from the corresponding author. TPM, DESeq2, and DEG values for each individual donor pool and data for modules can be downloaded from http://www.endothelial-response.org/. All gene expression profile plots and data for all genes can be downloaded directly from the Web site. The data are available as TPM or DEG FC values and can be split by sex and filtered by observation timeframes of interest.

## Results

Existing studies of EC responses to inflammatory stimuli, such as TNF, focus primarily on early signaling and associated transcriptional changes. In the present study, we profiled global changes in the EC transcriptome following TNF stimulation over an extended time course. HUVECs were extracted and pooled by donor sex before treatment with or without TNF for 0.5, 1, 2, 4, 6, 8, 12, 24, 36, 48, or 72 h and subsequent analysis by RNA sequencing (Supplemental Fig. 1A).

### TNF induces EC transcriptome modifications over an extended time course

**FIGURE 1.**
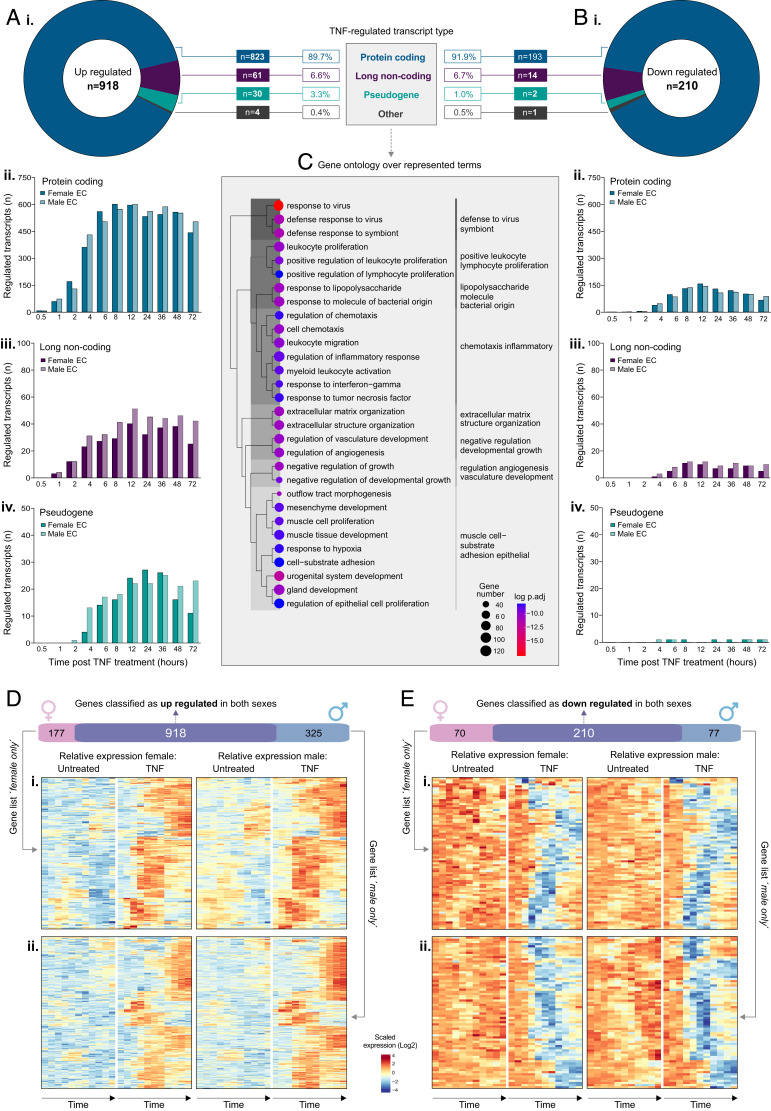
Overview of TNF-regulated genes in human endothelial cells. HUVECs (ECs, *n* = 5) were treated with or without TNF-α and harvested at 0.5, 1, 2, 4, 6, 8, 12, 24, 36, 48, or 72 h before RNA-sequencing analysis. Genes were classified as (**A**) upregulated or (**B**) downregulated by TNF (FC versus untreated control >2 or <0.5, respectively). Plots show (**i**) total TNF-regulated genes (in both sexes) and corresponding biotype and the number of (**ii**) protein coding, (**iii**) lncRNA, or (**iv**) pseudogenes regulated at each time point, by sex. (**C**) GO analysis showing overrepresented terms for all TNF-regulated transcripts. (**D** and **E**) The number of genes classified as either upregulated or downregulated, respectively, in females or males only or in both sexes. Heatmaps show relative expression in control or TNF-treated female (left) or male (right) samples for genes reaching the threshold for classification as TNF regulated in (**i**) males or (**ii**) females only.

Average DESeq2 expression values for each treated sample (biological replicates: *n* = 3 male and *n* = 2 female) were normalized to the sex- and time-matched untreated controls to identify DEGs. Totals of 2100 positive and 2162 negative DEGs were identified (FC versus untreated control, FC log_2_ >1 and FC log_2_ < −1, respectively [in both male and female sample sets]; TPM >1 at least one sample time point; raw counts >10 in all samples, adjusted by *p* value) (Supplemental Fig. 1B and *Materials and Methods* for analysis details; Supplemental Table II). Of these, a total of 918 genes were further classified as upregulated in response to TNF (Fig. 1Ai), and 210 were classified as downregulated (Fig. 1Bi), based on a TNF-induced change from a relatively stable baseline expression in untreated (control) ECs over time (CV between TPM values <0.3) (Supplemental Fig. 1Ci, 1Di). Genes classified as DEGs due to changes in expression in untreated control ECs over time (Supplemental Fig. 1Cii, 1Diii) or a TNF-induced lag in such changes (Supplemental Fig. 1Dii) were excluded because such changes may be linked to in vitro culture conditions and/or the influence of TNF on other inflammation-independent temporal processes.

The majority of upregulated genes were classified as protein coding (823 of 918; 89.7%), followed by long noncoding RNAs (lncRNAs) (61 of 918; 6.6%), pseudogenes (30 of 918; 3.8%), and “others” (three TEC, one sRNA) (Fig. 1Ai). A similar profile was observed for downregulated transcripts, with the majority classified as protein coding (193 of 210; 91.9%), followed by lncRNA (14 of 210; 6.9%), pseudogenes (2 of 210; 1%), and “others” (one TEC, one sRNA) (Fig. 1Bi). We performed GO analysis (35) to identify overrepresented groups in genes classified as regulated by TNF; significant enrichment terms included “immune system processes” (false discovery rate [FDR] 9.5 × 10^−44^)” and “response to cytokine” (FDR 8.2 × 10^−39^) (Supplemental Table III; overall summary in Fig. 1C).

To investigate the temporal profile of genes up- or downregulated in response to TNF, we determined the number of transcripts classified as such at each time point in each sex (Fig. 1Aii–1Aiv, 1Bii–1Biv). A limited number of protein coding transcripts (*n* = 10) were classified as upregulated already by 30 min after stimulation in both sexes (Fig. 1Aii), including those encoding for components of the NF-κB signaling pathway (*NFKBIA*, *NFKBIZ*), chemokines regulated by this pathway (*CXCL2*, *CXCL3*, *IL6*), and *FAM167A*, which was recently described as an activator of the noncanonical NF-κB pathway in chronic myeloid leukemia (43) (Supplemental Table III).

A small panel of lncRNAs were classified as upregulated by 1 h after stimulation in both sexes (*n* = 10) (Fig. 1Aiii), including those known to be expressed in response to inflammation in ECs, such as *MIR155HG* (44), but also those not previously described in this context, such as wound and keratinocyte migration-associated lncRNA 2 (*WAKMAR2*), which restricts inflammatory chemokine production in keratinocytes and enhances their migratory capacity (45).

TNF-responsive pseudogenes tended to be classified as upregulated slightly later in the time course than protein coding or long noncoding genes. From a total of 30 pseudogenes classified as upregulated by TNF (Fig. 1Aiv), 10 (33%) of 30 were members of the ferritin gene family (*FTH1P2*, *7*, *8*, *10*, *11*, *12*, *15*, *16*, *20*, *23*), some of which have known regulatory functions (46). Ferritin has a role in EC angiogenesis (47) and chemokine signaling (48); a potential role of this family in the EC response to inflammatory stimulation remains to be explored. Overall, the total number of genes that were upregulated in response to TNF was highest between 12 and 24 h after stimulation (Fig. 1Aii, 1Aiii, and 1Aiv).

In contrast to TNF-induced upregulated genes, no protein coding genes were classified as downregulated at the earliest time point (Fig. 1Bii). Those classified as such within the first 4 h (*n* = 54) included *HOXA9*, which inhibits NF-κB–dependent EC activation (49). The total number of downregulated protein coding transcripts peaked around 12 h (Fig. 1Bii). lncRNAs classified as downregulated in response to TNF were also classified as such later than those that were upregulated (Fig. 1Biii), and only a small number of pseudogenes were consistently downregulated across the time course (Fig. 1Biv).

Thus, the global primary EC response to TNF stimulation consists predominantly of the induction of protein coding and, to a lesser extent, long noncoding gene expression.

#### TNF-induced changes are comparable between male and female endothelial cells

Differences in inflammatory response have previously been described in female and male ECs (22, 50). In our analysis described above, 177 genes were classified as upregulated only in female samples and 325 only in male samples (Fig. 1D), and 70 genes were classified as downregulated only in female samples and 77 only in male samples (Fig. 1E, Supplemental Table I). Heatmap plots of genes classified as up- or downregulated only in female ECs (Fig. 1Di and 1Ei, respectively) or only in male ECs (Fig. 1Dii and 1Eii, respectively) revealed similar patterns of regulation over the time course in the other respective sex (relative expression in female ECs on the left and male ECs on the right). Thus, these differences in classification were likely due to the strict thresholding criteria we initially applied to identify the most consistently and strongly regulated genes rather than to a fundamental difference between the responsiveness of ECs from each sex. Indeed, when we applied a threshold of up- or downregulation in one or more samples of one sex (FC log_2_ abs >1) versus no regulation in any samples of the other sex (FC abs <1.1 [FC log_2_ <0.1375]), no genes were classified as sex-specifically regulated by TNF. MDS revealed high global similarity in TNF-induced transcriptome modifications over the time course in female (Supplemental Fig. 2Ai) and male (Supplemental Fig. 2Aii) ECs. Thus, the chromosomal composition of the EC does not appear to markedly effect the global transcriptional response to TNF stimulation.

To identify baseline (unstimulated) differences in gene expression in female versus male ECs, we performed differential expression analysis between the control unstimulated samples of each sex. Ninety-nine genes were classified as differentially expressed between the sexes at every time point (FC log_2_ abs >1, adjusted *p* < 0.05), with 58 genes being higher in females and 41 being higher in males (Supplemental Fig. 2B). As expected, Y chromosome genes represented the most significantly differentially expressed genes in male ECs (Supplemental Fig. 2Bi), and the lncRNA *XIST*, a regulator for X inactivation (51), was the most significantly differentially expressed gene in female ECs (Supplemental Table III). Of the non–sex chromosome–linked genes that were differentially expressed between male and female ECs at baseline (with mean expression [in either sex] TPM >1), 14 were also classified as TNF regulated in both sexes (Supplemental Fig. 2Bii and Supplemental Table III). Of these, nine were more highly expressed in female than in male cells (*PLLP*, *TNFSF10*, *CBLN3*, *MMP12*, *ANGPT2* [upregulated by TNF], and *PLXDC2*, *ABCA8*, *MS4A2*, *AC104083.1* [downregulated by TNF]) (Supplemental Fig. 2Bii), and five were more highly expressed in male than in female cells (*FOSB*, *IL27RA*, *CDH2*, *PHLDA2* [upregulated by TNF] and *PITPNM3* [downregulated by TNF]) (Supplemental Fig. 2Bii). Although such genes had similar TNF regulatory profiles in female and male cells (for examples, see Supplemental Fig. 2Biii and 2Biv), the differences in absolute expression levels could indicate sex-specific transcriptional regulation.

#### WGNCA reveals TNF-induced gene signatures

**FIGURE 2.**
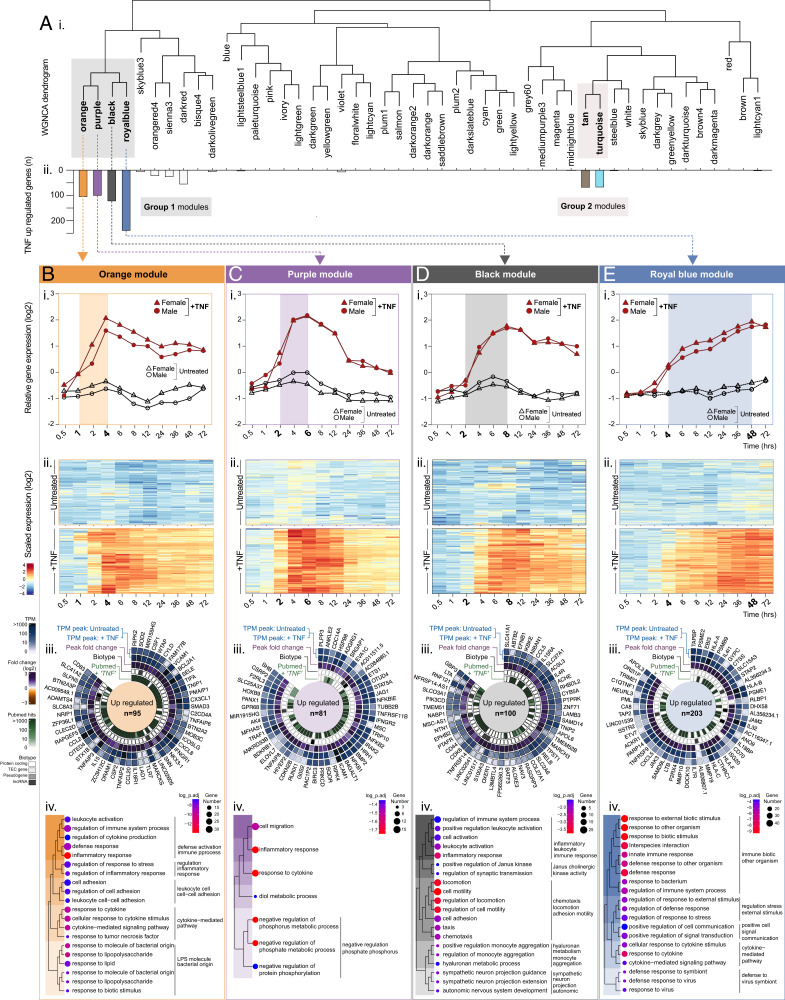
WGNCA reveals temporal relationships between TNF upregulated genes: Group 1 “early induced.” HUVECs (ECs, *n* = 5) were treated with or without TNF-α and harvested at 0.5, 1, 2, 4, 6, 8, 12, 24, 36, 48, or 72 h before RNA-sequencing analysis. WGNCA was used to cluster genes into modules, based on expression pattern similarity across sample sets. (**A**) (**i**) Dendrogram showing WGNCA modules and (**ii**) corresponding distribution of genes previously classified as TNF upregulated. For modules (**B**) orange, (**C**) purple, (**D**) black, (**E**) and royal blue, (**i**) relative gene expression plots displaying the module eigengenes and (**ii**) heatmaps showing the temporal expression profile for all genes in the module with eigengene >0.8 (*p* < 0.05). (**iii**) Circle plots for the top 50 genes classified as TNF upregulated within each module, showing expression values in control and stimulated ECs, the peak FC, the biotype and number of PubMed hits for “gene name” + “TNF,” and (**iv**) overrepresented terms by GO analysis (biological processes).

To explore the potential relationship between TNF-regulated genes in terms of expression dynamics over time, we performed a WGNCA (34), where correlation coefficients between all transcripts across the sample set (male and female samples were handled together) were calculated and subsequently clustered into 48 related modules (Fig. 2Ai), based on expression profile similarity. In addition to the identification of coregulated genes, this analysis could potentially highlight genes with a currently unknown role in specific stages of the EC response to TNF.

##### TNF upregulated genes have two main regulation profiles

The majority of genes we earlier classified as upregulated in response to TNF stimulation fell into two main module regions on the WGNCA dendrogram (Fig. 2Ai), occupying neighboring leaves on common clades: annotated as group 1 (orange, purple, black, and royal blue) and group 2 (tan and turquoise) modules (Fig. 2Aii, see shaded boxes).

#### Upregulated group 1: early induction (1–4 h after stimulation)

Group 1 modules contained genes that were upregulated by 1 h (orange) (Fig. 2Bi, 2Bii), 2 h (purple, black) (Fig. 2Ci, 2Cii, 2Di, 2Dii) or 4 h (royal blue) (Fig. 2 Ei, 2Eii) after TNF stimulation. Genes in the orange (Fig. 2Bi, 2Bii), purple (Fig. 2Ci, 2Cii), and black (Fig. 2Dii) modules reached a peak between 4 and 8 h, after which the differential expression versus control gradually declined. Several genes with well-established roles in the initial stages of inflammation were in the “earliest responder” orange module (see Fig. 2Biii for the top 50 upregulated genes with highest correlation to the eigengene), including those encoding for components of the NF-κB signaling pathway, such as *NFKBIA*, *NFKBIZ*; leukocyte adhesion receptors, such as *VCAM1* and *SELE*; and various chemokines or cytokines, such as *CXCL8*, *CX3CL1*, *CCL2*, and *CCL20*. A PubMed search retrieving the number of publications containing the search terms “gene” and “TNF” revealed that most of the orange module top 50 genes had previously been referred to in the context of the TNF response (Fig. 2Biii). However, some, such as *FAM177B*, an uncharacterized gene with little to no expression under baseline conditions, have no previous reported link with the EC response to TNF or indeed inflammation processes in general. This was also true for some genes in the other modules, such as purple module long noncoding gene *MIR1915HG*, which currently has no assigned function, and black module gene *ALOXE3*, which encodes for a member of the lipoxygenase family that was recently reported as expressed in ECs and upregulated by shear stress exposure (52). One could speculate that such genes encode for proteins with currently unknown important roles in the initial stages of inflammation and thus represent interesting candidates for functional investigation. GO analysis of TNF upregulated genes in the orange (Fig. 2Biv), purple (Fig. 2Civ), and black (Fig. 2Div) modules, revealed overrepresentation of similar inflammation-related terms, such as “inflammatory response” and “response to cytokine” (Supplemental Table III). Apoptosis-related GO groups were overrepresented in the orange module only (apoptotic processes [FDR 1.18 × 10^−5^]; Supplemental Table III). Upregulated genes in these groups included those encoding for proteins that inhibit apoptosis, such as *TNFAIP3*, *SOD2*, *NUAK2*, and *BCL2A1*, as well as those with a likely role in the induction of cell death, such as *CYLD* and *PMAIP1*, and those with potential roles in both, depending on context, such as *CD40*. Outside of the orange module, other genes with antiapoptotic and prosurvival functions, such as *XIAP*, *BCL2A1*, *BIRC3*, and *CFLAR*, were also upregulated by TNF (Supplemental Table II). Together, this suggests that TNF induces both pro- and antiapoptotic factors with complex interactions, consistent with the concept that death is not the default cellular response to TNF and that various protectives brakes, or cell death checkpoints, protect against apoptosis (53).

In contrast to the other group 1 modules, TNF upregulated genes in the royal blue module remained elevated across the 72-h time course (Fig. 2Ei, 2Eii) and contained genes encoding for several types of pattern recognition receptors, such as TLRs (*TLR2*, *TLR5)*, RIG-I-like receptors (*DDX58*, *DHX58*, *IFIH1)* and NOD-like receptors (*NOD2*, *NLRC5*), and cyclic GMP-AMP synthase (*cGAS*). This module also contained *ISG20*, an ISG that encodes a nuclease enzyme that can cleave viral RNA. GO analysis of TNF upregulated genes in this module revealed that overrepresented terms included those linked to viral defense, such as response to virus (Fig. 2Eiv, Supplemental Table III).

#### Upregulated group 2: delayed induction (12–24 h after stimulation)

**FIGURE 3.**
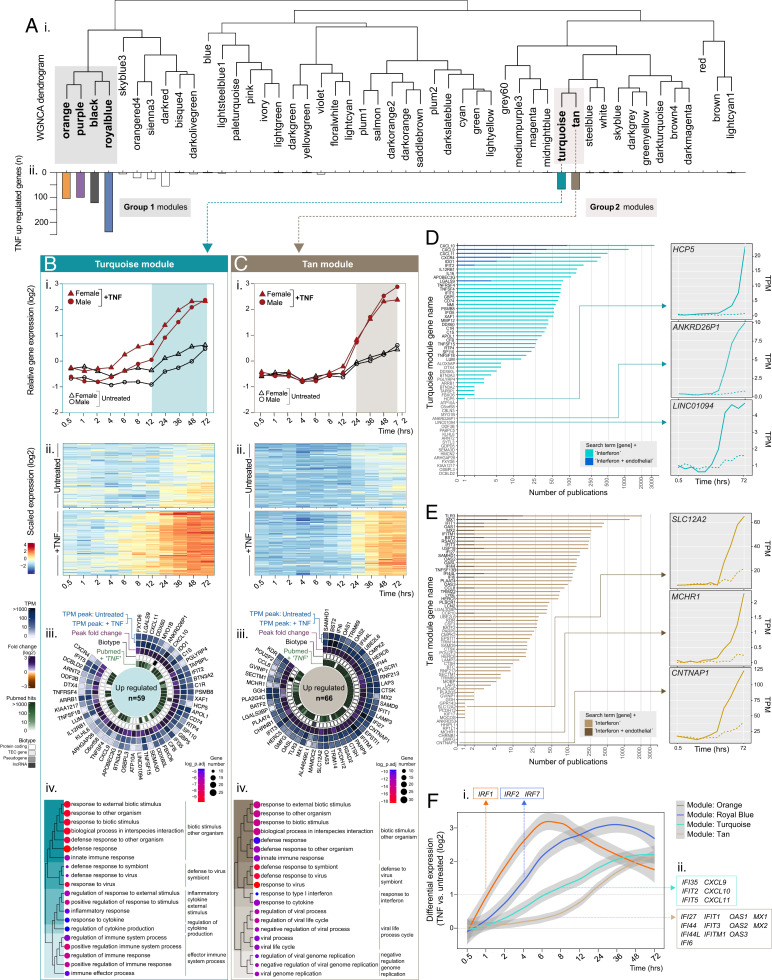
WGNCA reveals temporal relationships between TNF upregulated genes: Group 2 “delayed induced.” HUVECs (ECs, *n* = 5) were treated with or without TNF-α and harvested at 0.5, 1, 2, 4, 6, 8, 12, 24, 36, 48, or 72 h before RNA-sequencing analysis. WGNCA was used to cluster genes into modules, based on expression pattern similarity across sample sets. (**A**) (**i**) Dendrogram showing WGNCA modules and (**ii**) corresponding distribution of genes previously classified as TNF downregulated. For modules (**B**) tan and (**C**) turquoise, (**i**) relative gene expression plots displaying the module eigengenes and (**ii**) heatmaps showing the temporal expression profile for all genes in the module with correlation to the eigengene >0.8 (*p* < 0.05). (**iii**) Circle plots for the top 50 genes classified as TNF upregulated within each module, showing expression values in control and stimulated ECs, the peak FC, the biotype and number of PubMed hits for “gene name” + “TNF,” and (**iv**) overrepresented terms by GO analysis (biological processes). (**D** and **E**) The number of hits returned for TNF upregulated genes in the tan or turquoise modules, respectively, in a PubMed search for “gene name” + “IFN” and “gene name” + “IFN” + “endothelial,” with temporal expression plots for selected examples (created using the Web site tool provided as part of this study). (**F**) Temporal distribution of IFN-related genes upregulated by TNF across orange, royal blue, tan, and turquoise modules.

Group 2 modules (turquoise and tan) (Fig. 3A) contained genes that were upregulated between 12 and 24 h after TNF treatment, with the highest differential expression versus control at 72 h (Fig. 3Bi, 3Bii, 3Ci, 3Cii). Genes that were upregulated following TNF treatment in the turquoise module (Fig. 3B) included those encoding for a panel of IFN-induced cytokine ligands for the APC receptor CXCR3: *CXCL9*, *CXCL10*, and *CXCL11* (Fig. 3Biii), the expression of which is linked with viral infection, progression, and replication control (54–56). Other ISGs in this module included IFIT2, IFIT5, and IFIT35, that, to our knowledge, have not previously been reported as being modified following TNF stimulation. GO analysis of upregulated genes in the turquoise module revealed overrepresentation of terms associated with defense to virus symbiont (Fig. 3Biv), in addition to general inflammation terms, such as response to cytokine (Supplemental Table III). Genes that were upregulated following TNF treatment in the tan module also included a large panel of ISGs, such as, among others, *IFIT1*, *IFI6*, *IFI27*, *OAS1/2/3/L*, *MX1/2*, and *IFITM1* (Fig. 3Ciii), and, correspondingly, GO analysis revealed a highly significant overrepresentation of terms related to response to IFN and viral infection (Fig. 3Civ, Supplemental Table III). Thus, this latter stage of the response was dominated by the induction of IFN-/antiviral-related gene transcription. Indeed, a PubMed search for studies citing genes that were upregulated following TNF treatment in the turquoise (Fig. 3D) and tan (Fig. 3E) modules, together with the term “IFN,” revealed that most had been reported previously in this context (40 of 59 [68%] and 57 of 66 [88%], respectively). Additional searches using related terms (“virus,” “viral,” “antiviral,” “IFN stimulated gene,” or “immune response”) showed a similar distribution of hits (Supplemental Table III). Although not all hits containing two terms necessarily imply a meaningful functional association, such an analysis can offer a perspective regarding the likelihood of the absence or presence of a link. Only a small proportion of the genes had PubMed hits that included the terms “IFN” and “endothelial” (Fig. 3D and 3E, dark shaded bars) or “viral” and “endothelial” (Supplemental Table III), indicating that these pathways are less well understood in this cellular context. Genes in the turquoise module with only one or no hits linking them to IFN (Fig. 3D) included several noncoding genes, which are typically less well studied than protein coding genes, such as *HCP5*, which has polymorphisms linked to HIV viral load (57) and is a susceptibility locus for Kawasaki disease, a systemic vasculitis of infants and children (58), the uncharacterized pseudogene *ANKRD26P1*, and *LINC01094* (Fig. 3D, right panels). Thus, it is possible that such genes have currently unknown roles in EC IFN-related signaling, based on the similarity of their temporal expression profile with others in the module.

Eleven genes in the tan module had only one or no hits linking them to IFN, including the solute transported *SLC12A2*, the G protein–coupled receptor *MCHR1*, and the paranodal junction component *CNTNAP1* (Fig. 3E, right panel). The role of these genes in inflammation and any possible connection to EC IFN and/or viral response signaling remain to be established.

### ISG expression following TNF treatment is not driven by de novo IFN production

**FIGURE 4.**
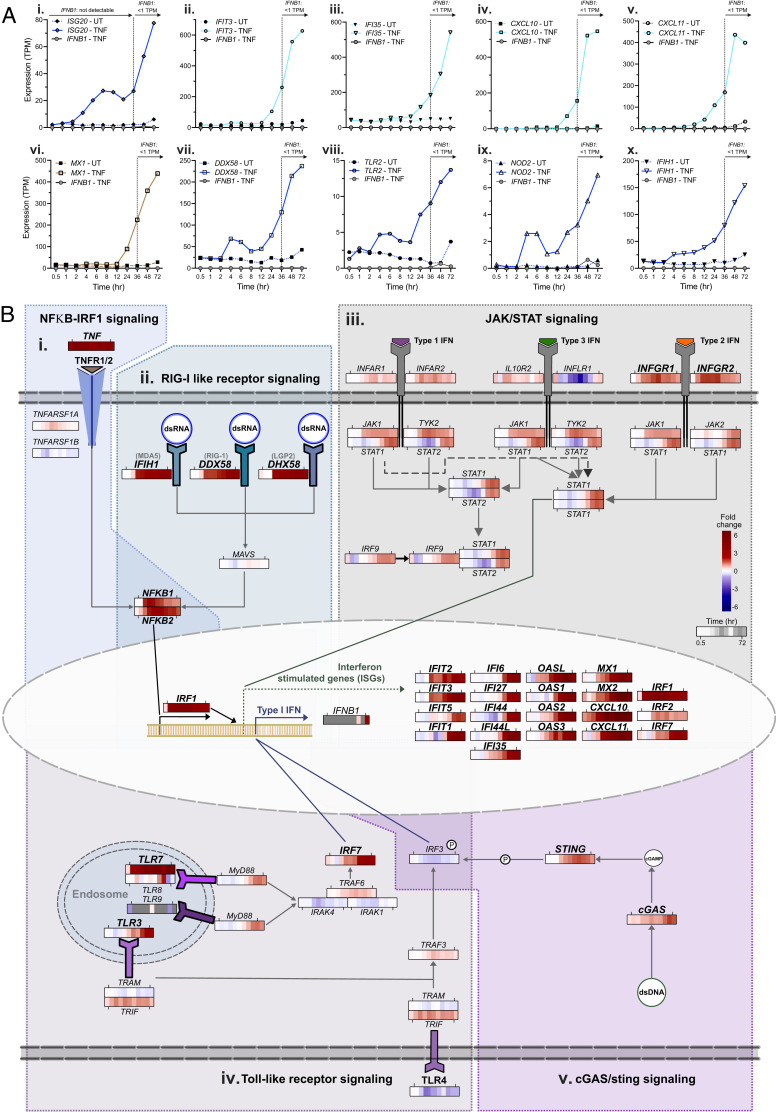
TNF upregulation of IFN-stimulated and pattern recognition receptor gene expression is independent of de novo IFN production. (**A**) Temporal expression profiles in unstimulated control or TNF-α–stimulated ECs (*n* = 6) for *IFNB1* and (**i**) *ISG20*, (**ii**) *IFIT3*, (**iii**) *IFI35*, (**iv**) *CXCL10*, (**v**) *CXCL11*, (**vi**) *MX1*, (**vii**) *DDX58*, (**viii**) *TRL2*, (**ix**) *NOD2*, and (**x**) *IFIH1* (sample set F). (**B**) Summary of key genes and pathways linked to IFN-stimulated gene expression: (**i**) NFKB-IRF1 signaling (94), (**ii**) RIG-I-like receptor signaling (95), (**iii**) JAK-STAT signaling (96), (**iv**) TLR signaling (97), and (**v**) cGAS-STING signaling (95). Heatmaps show the differential gene expression between unstimulated control and TNF-stimulated ECs for the adjacent gene. Gray squares in the heatmap are the result of zero TPM values; thus, differential expression is not calculated. Bold gene symbols denote those that were classified as TNF upregulated. Heatmaps were created using the Web site tool provided as part of this study.

Although the mechanisms of ISG expression following TNF treatment of ECs are not well understood, previous studies have reported that it is driven by de novo production and subsequent autocrine signaling of type I IFN (IFN-β) (9). We found that three of the nine members of the IFN regulatory factor family (IRF1–IRF9), which are critical for the induction of type I IFN (59), were upregulated following TNF stimulation, and all were found in the group 1 “early responding” orange (*IRF1*) and royal blue (*IRF2* and *IRF7*) modules (Fig. 3Fi), thus temporally preceding the induction of the majority of the ISGs, which fall in modules turquoise and tan (Fig. 3Fii). However, only a modest induction of *IFNB1* was observed at later time points (maximum any sample, any time point = 1.02 TPM [24 h]) (mean values for all IFN proteins in Supplemental Table III); importantly, expression of many ISGs preceded the time point at which *IFNB1* was expressed at detectable levels, such as *ISG20* (Fig. 4Ai), *IFIT3* (Fig. 4Aii), *IFI35* (Fig. 4Aiii), *CXCL10* (Fig. 4Aiv) *CXCL11* (Fig. 4Av), and *MX1* (Fig. 4Avi). The same was also observed for various pattern recognition receptors, such as *DDX58* (Fig. 4Avii), *TLR2* (Fig. 4Aviii), *NOD2* (Fig. 4Aix), and *IFIH1* (Fig. 4Ax). Thus, in this system, the transcriptional induction of such genes was not driven by autocrine IFN-β signaling. Changes in the expression of genes encoding for key components of the IFN/antiviral response pathways (JAK/STAT, NF-κB-IRF1, TLR, RLR, and cGAS/cyclic GMP-AMP receptor stimulator of IFN genes [STING]) following TNF treatment are summarized in Fig. 4B.

#### TNF downregulated genes have two main regulation profiles

**FIGURE 5.**
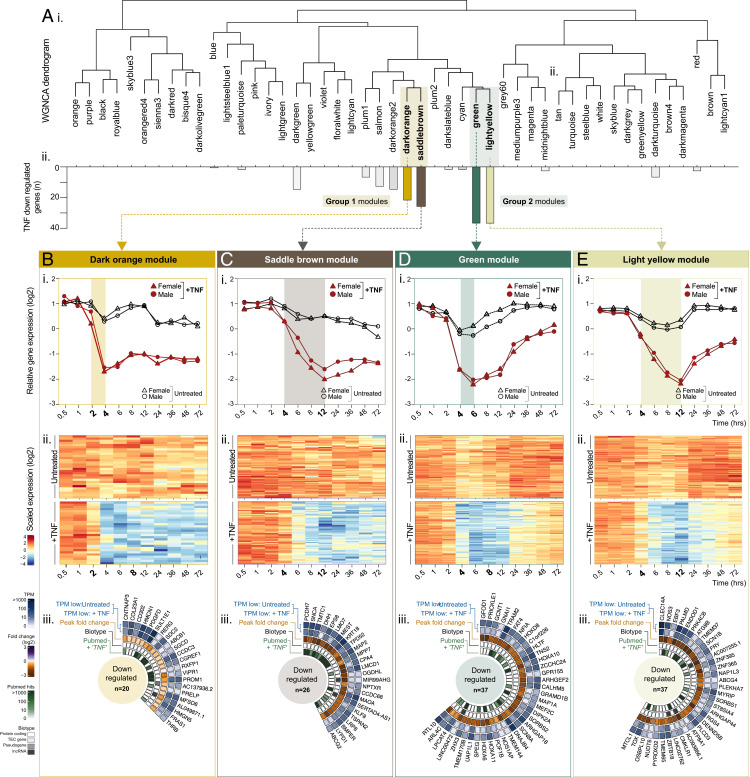
WGNCA reveals temporal relationships between TNF-downregulated genes. HUVECs (ECs, *n* = 5) were treated with or without TNF-α and harvested at 0.5, 1, 2, 4, 6, 8, 12, 24, 36, 48, or 72 h before RNA-sequencing analysis. WGNCA was used to cluster genes into modules, based on expression pattern similarity across sample sets. (**A**) (**i**) Dendrogram showing WGNCA modules and (**ii**) corresponding distribution of genes previously classified as TNF downregulated. For modules (**B**) dark orange, (**C**) saddle brown, (**D**) green, and (**E**) light yellow: (**i**) relative gene expression plots displaying the module eigengenes and (**ii**) heatmaps showing the temporal expression profile for all genes in the module with correlation to the eigengene >0.8 (*p* < 0.05). (**iii**) Circle plots for genes classified as TNF downregulated within each module, showing expression values in control and stimulated ECs, the peak FC, and the biotype and number of PubMed hits for “gene name” + “TNF.”

The majority of genes classified as downregulated in response to TNF stimulation fell into two main regions on the WGNCA dendrogram (Fig. 5Ai), occupying neighboring leaves on common clades: annotated as group 1 (dark orange, saddle brown) and group 2 (green and light yellow) modules (Fig. 5Aii) (highlighted with shaded boxes). Modules in both groups contained genes that were downregulated by TNF between 2 and 4 h after stimulation (Fig. 5Bi, 5Bii, 5Ci, 5Cii, 5Di, 5Dii, 5Ei, 5Eii).

##### Downregulated group 1: inhibition maintained over time

Genes in the group 1 dark orange (Fig. 5Bi, 5Bii) and saddle brown (Fig. 5Ci, 5Cii) modules reached maximum downregulation 4 and 12 h after stimulation, respectively, an effect that was maintained across the remainder of the time course. There were no significantly enriched GO terms in lists of downregulated genes appearing in the group 1 downregulated modules, possibly due to the low numbers or lack of previous reports of gene function.

##### Downregulated group 2: inhibition resolved over time

In contrast to group 1, TNF downregulated genes in group 2 modules, green (Fig. 5Di, 5Dii) and light yellow (Fig. 5Ei, 5Eii), trended back toward baseline level after reaching maximum downregulation at 6 and 12 h after stimulation, respectively. Again, there were no significantly enriched GO terms in any of lists of downregulated genes appearing in the highlighted modules; indeed, an automated PubMed search for the search terms “downregulated gene” and “TNF” retrieved markedly fewer hits than the equivalent search for genes we previously classified as upregulated by TNF (Fig. 5B–Eiii). Despite a lack of significant enrichment terms, some links between constituent genes could be observed; for example, four out of five TNF downregulated homeobox family transcription factor genes were classified into module green (*HOXA6*, *HOXA10*, *HOXA11*, and *HOXD8*) (Fig. 5Diii). Although *HOX10* has been reported as an activator of canonical NF-κB signaling in pancreatic cancer cells (60), and although the antisense to *HOXA11* (*HOXA11-AS*) was linked to protection of EC barrier function following injury (61), insight into the potential role of these genes in the EC TNF response is currently lacking.

In both TNF downregulated modules groups 1 and 2, gene baseline expression levels and TNF response profiles were similar between male and female samples (Fig. 5Bi–5Ei and Supplemental Fig. 2C).

### Visualization of temporal gene regulation pathways using the Web site tool

We have created a Web site resource that allows users to perform both gene-centric and module-based lookup of our endothelial TNF time-course data. Key features include a data viewer to observe the transcriptional responses of specific genes (Supplemental Fig. 3A) or the WGNCA module into which they were classified (Supplemental Fig. 3C) and the generation of vector-image downloadable expression plots for both predefined and custom gene lists, such as TNF-regulated CXCL and CCL chemokines (Supplemental Fig. 3B). The dataset can also be analyzed to identify genes with high correlation across conditions with any given input gene (Supplemental Fig. 3D).

## Discussion

In the present study, we used RNA sequencing to measure the temporal response of ECs to TNF stimulation, incorporating 11 time points up to 72 h. Following the identification of TNF-regulated genes, we used WGNCA to understand the global temporal context of these transcriptomic changes. To our knowledge, this is the first study to map the EC TNF response at a transcriptome-wide level in such temporal detail.

We identified two main profiles into which TNF-induced genes could be classified: those with an “early” or “delayed” induction. Early induced genes (∼1–4 h after stimulation) included many previously well studied in this context, such as those encoding for EC leukocyte adhesion receptors (e.g., *SELE*, *VCAM1*, and *ICAM1*) (62), but we also identified several genes with similar expression dynamics that encoded for completely uncharacterized proteins (e.g., *FAM177B*), which could be interesting candidates for future study in the context of inflammation. TNF upregulated genes with a delayed induction (12–24 h after stimulation) were primarily ISGs, whose regulation by inflammatory cytokines in ECs is generally not well understood, but our observations were consistent with one recent study that reported the induction of a late-stage IFN response in ECs following TNF stimulation (63). ISG expression is considered to be driven primarily by the production and subsequent signaling of IFN via canonical (JAK-STAT) or noncanonical pathways (64).

We found that three of the nine members of the IFN regulatory factor family (*IRF1*, *2*, and *7*), which are critical for the induction of IFN (59), were upregulated by TNF in ECs at time points that preceded ISG expression. Of these, *IRF1* and *IRF7* have been implicated as positive regulators of type I IFN production (65), and previous reports have shown that TNF-induced expression of ISGs, such as *CXCL9* and *CXCL10*, in murine ECs was dependent on *IRF1*-induced de novo production of IFNβ (*IFNB1*) and its subsequent autocrine signaling through STAT1 (9). However, our data indicated that EC expression of ISGs following TNF treatment was independent of de novo production of IFN, because we did not observe its transcription prior to ISG expression.

We observed an upregulation of genes encoding for cGAS and the STING, a system that detects pathogenic DNA (66). A recent study showed that the expression of various ISGs that were induced by TNF in fibroblasts, including *CXCL10*, *IFIT1*, and *IFIT44* (all of which were also upregulated by TNF in the present study), was markedly reduced in cGAS and STING knockout cells (67). TNF-dependent mitochondrial damage and mtDNA leakage were shown to underlie this response; one could speculate that a similar mechanism contributes or occurs in ECs.

We observed an upregulation of genes encoding for other pattern recognition receptors, including TLRs (*TLR2*, *TLR5)*, RIG-I-like receptors (*DDX58*, *DHX58*, *IFIH1*), and NOD-like receptors (*NOD2*, *NLRC5*). Although these receptors are known to induce production of IFN and subsequent expression of ISG in response to bacterial or viral ligands (68, 69), whether they have a role in the induction of ISGs in ECs following TNF production, similar to that reported for cGAS and STING (67), remains to be explored.

We identified several noncoding RNAs within the gene modules that otherwise predominantly contained ISGs, including ENSG00000225886 (antisense to *IFI6*); *NRIR*, a negative regulator of SARS-CoV-2 infection (70); and *LINC02056*, an IFN-inducible transcript with a proposed role in IRF3 nuclear translocation (71). As is often the case with noncoding genes, functional annotation of others was lacking, such as *LINC02051* and *LINC02068*; these are potentially interesting candidates to study in the context of the EC IFN response. Overall, deciphering the relative contributions of various pathways in the TNF-induced expression of ISGs is complex, with potential differences between cell types and species.

We identified a panel of 210 gene transcripts that were at lower levels following TNF stimulation. Although studies of the TNF response tend to focus on genes whose expression is increased in response to stimulation, several of those we identified as downregulated had been reported previously as such, including *NOS3*, the mRNA stability of which is inhibited by TNF (72); *DHH*, which prevents EC activation (73); and *RGS4*, which regulates the secretion of von Willebrand factor (74). However, many had not been reported previously in this context, such as *CNR1*, *SLC7A8*, and *CLEC14A*, which were among the most downregulated by TNF.

Sex differences have been reported in several inflammatory conditions of the vasculature, such as cardiovascular disease (75) and thrombosis (76). Although our data indicate that chromosomal composition alone does not markedly affect the EC response to TNF, a multitude of other factors influence vascular responses in vivo, such as sex hormones, which may drive sex-linked inflammatory differences (77, 78).

### Study strengths and limitations

One of the main strengths of our study is the size of the dataset generated; we analyzed 130 samples, incorporating 33 biological replicates. The global EC transcriptome was analyzed at 11 different time points after TNF treatment, and the inclusion of matched control samples for each sample set at every time point allowed us to control for baseline transcriptional changes, such as those due to changes in the microenvironment (79) or cell density (80), which could otherwise be incorrectly annotated as TNF driven. To our knowledge, the Web site resource we provide (http://www.endothelial-response.org/) is the most extensive of its type; all data are accessible without the need for bioinformatic expertise.

The ECs we used in our study were isolated from human umbilical veins (HUVECs), from which we could generate a large amount of fresh primary ECs. Thus, we avoided the need to passage, freeze–thaw, or culture the ECs for a prolonged period prior to treatment and analysis, factors that could affect behavior and response to cytokines (81, 82). However, it should be acknowledged that this EC type is fetal rather than adult, and differences have been reported between the two, including the transcriptional response to TNF (83). Comparisons of microarray data for human dermal microvascular ECs (HMEC1) and HUVECs have shown that approximately half of the TNF-induced changes were specific for only one or the other of these EC types (83). However, key genes that were highlighted as only upregulated by TNF in HMEC1, but not HUVECs (e.g., *IL1B*, *DUSP6*, *OAS1*, *CLDN1*, *CD70*, *MMP12*), were classified as upregulated in our HUVEC dataset, potentially indicating that other factors, such as the sensitivity of EC types to passage in culture or freeze–thaw cycles, could influence response, as opposed to core characteristics of the EC types per se. Indeed, other studies show more similar characteristics between EC types, such as the response to shear stress exposure, which was largely comparable between human adult aortic cells and HUVECs (84). Furthermore, ECs have organ-specific heterogeneity (85, 86), meaning that vascular bed–specific responses to TNF might not be comparable to those we observed in HUVECs.

It should also be noted that ECs in our study are not cultured under flow or together with other cell types found in the normal microenvironment, both factors that can affect in vitro gene expression (87–90). Exposure to different levels of flow in vitro can modify the EC response to TNF (91), and thus our data may be more representative of EC responses in low rather than high shear–exposed vessels. Finally, although under steady-state conditions protein expression is highly dependent on mRNA level (92), the relationship between the two after state transitions, such as those induced by TNF, is subject to time-dependent processes, such as maturation, export, and translation of mRNA (92). Thus, there will be a delay between transcriptional changes and the associated protein level increase or decrease. Although extensive, our dataset does not provide a comprehensive overview of all aspects of the TNF response. The majority of microRNAs are not profiled because of the RNA isolation method used and the release of stored and secreted immediate responders, such as P-selectin and von Willebrand factor (93), and because processes such as protein phosphorylation and nuclear translocation are not measured.

## Supplementary Material

Supplemental 1 (PDF)

Supplemental 2 (XLS)

Supplemental 3 (XLS)

Supplemental 4 (XLS)
