# Supplementary material for: Global Transcriptome Analysis Reveals Distinct Phases of the Endothelial Response to TNF
Source: J Immunol. 2023 Nov 29;212(1):117–29. doi: 10.4049/jimmunol.2300419 (PMC10733583; doi:10.4049/jimmunol.2300419)
Supplement: Supplemental 1 (PDF) [file JI_2300419_Supplemental_1.pdf]

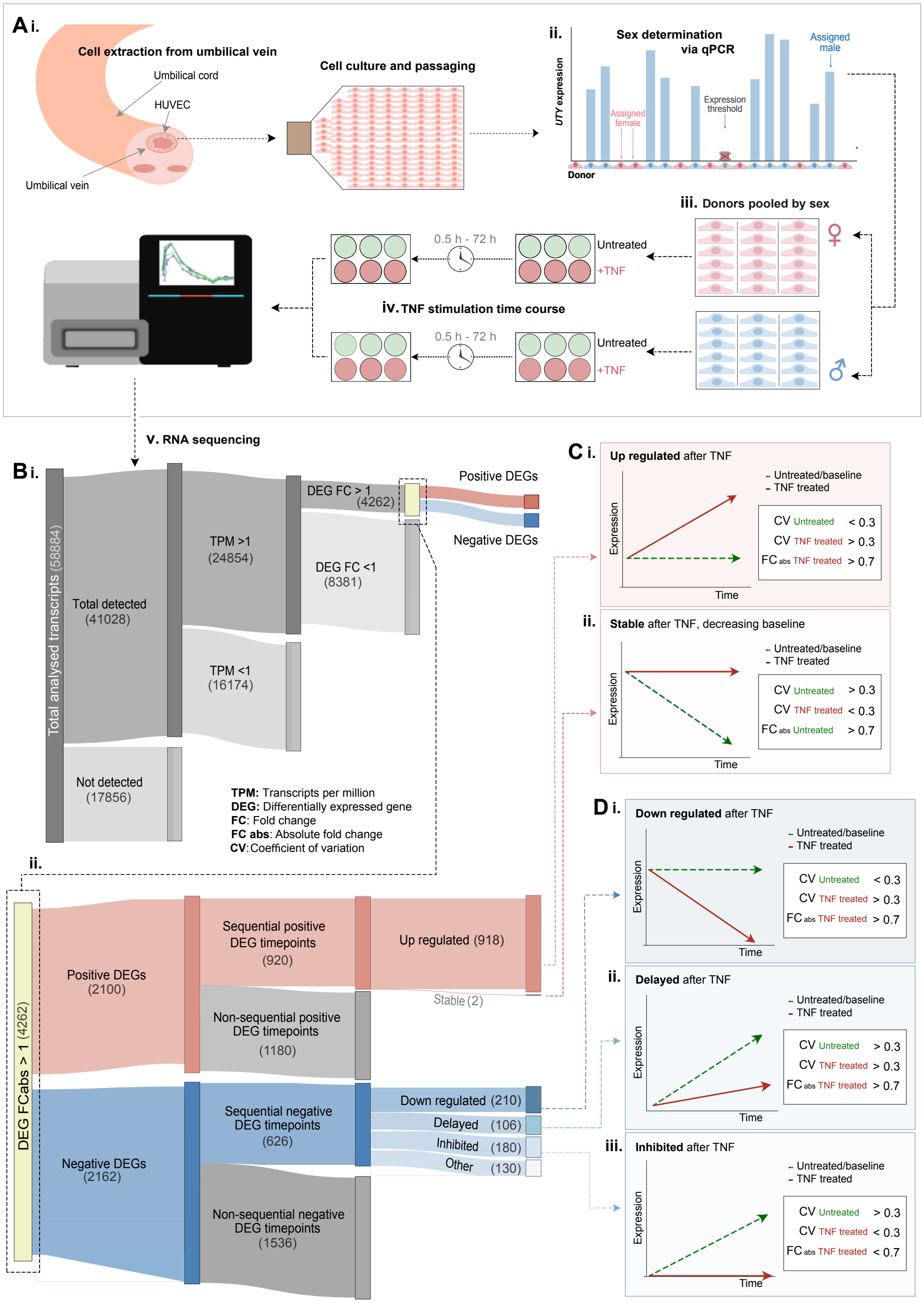

Figure S1. Experimental design and gene classifications. (**A**) Overview of the experimental procedure. (i) Endothelial cells were extracted from human umbilical veins and cultured to confluency before (ii) qPCR for Y-chromosome gene *UTY* was used to identify male vs. female donors. (iii) Cells were pooled into sex-matched sample sets, grown to confluency and (iv) stimulated with tumour necrosis factor alpha (TNF; 10 ng/mL), before RNA extraction at 0.5, 1, 2, 4, 6, 8, 12, 24, 36, 48 or 72 hours post stimulation, followed by (v) RNA sequencing analysis. (**B**) Sankey plots displaying (i) total number of genes detected and classified as differentially expressed genes (DEG) (ii) numbers of positive and negative DEG, and the subsequent classification as (**C**) Positive DEGs as: (i) *up regulated* by TNF from a stable baseline expression in control EC, or (ii) *stable* on the background of reduced baseline expression in control EC over time. (**D**) Negative DEGs as: (i) *down regulated* by TNF from a stable baseline expression in control EC, or (ii) *delayed* or (iii) *inhibited* by TNF, where baseline gene expression in control EC increases over time, but this change either occurs later or is not observed in TNF treated EC, respectively. Genes that did not fall into these groups were categorised as ´other´.

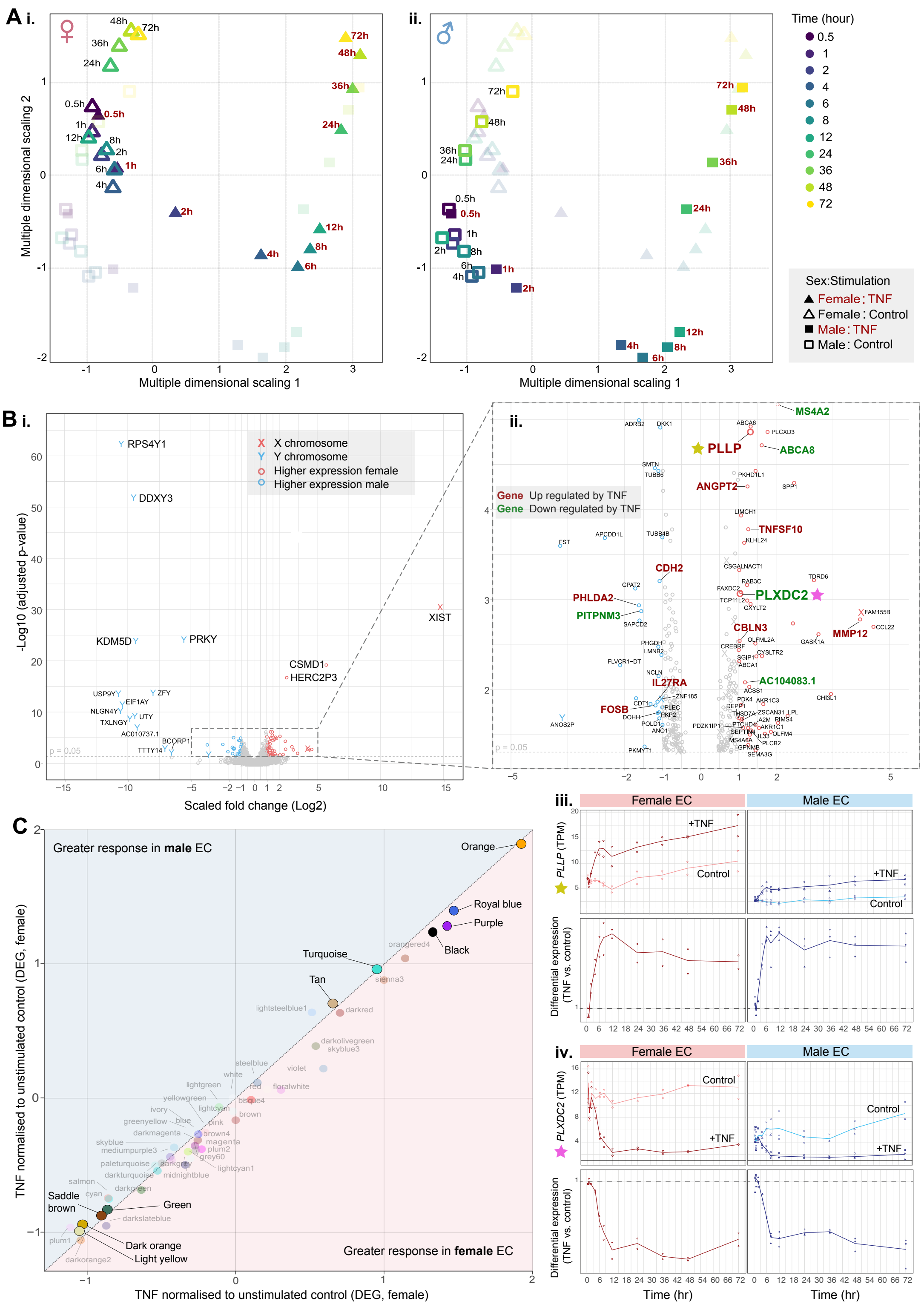

Figure S2. Sex-based comparison of global gene expression profiles. Human umbilical vein endothelial cells (EC, male n=3, female n=2) were treated with or without tumour necrosis factor alpha (TNF) and harvested at 0.5, 1, 2, 4, 6, 8, 12, 24, 36, 48 or 72 hrs, before RNAseq analysis. (**A**) Multidimensional scaling plot for control or TNF treated (i) female or (ii) male EC, at all analysed time points. (**B**) (i) Volcano plot displaying differentially expressed genes between male and female EC under baseline (unstimulated control) conditions, with X- and Y-chromosomal genes annotated with ´Y´ and ´X´, respectively (ii) panel showing all transcripts classified as sex-differentially expressed at baseline (mean expression [in either sex] TPM>1), with those also classified as up or down regulated by TNF highlighted in larger red or green text, respectively. Expression data is provided for example genes annotated with star symbols: (iii) *PLLP* and (iv) *PLXDC2*. (**C**) Scatter plot comparing normalised average expression values between male and female samples for each WGCNA module.

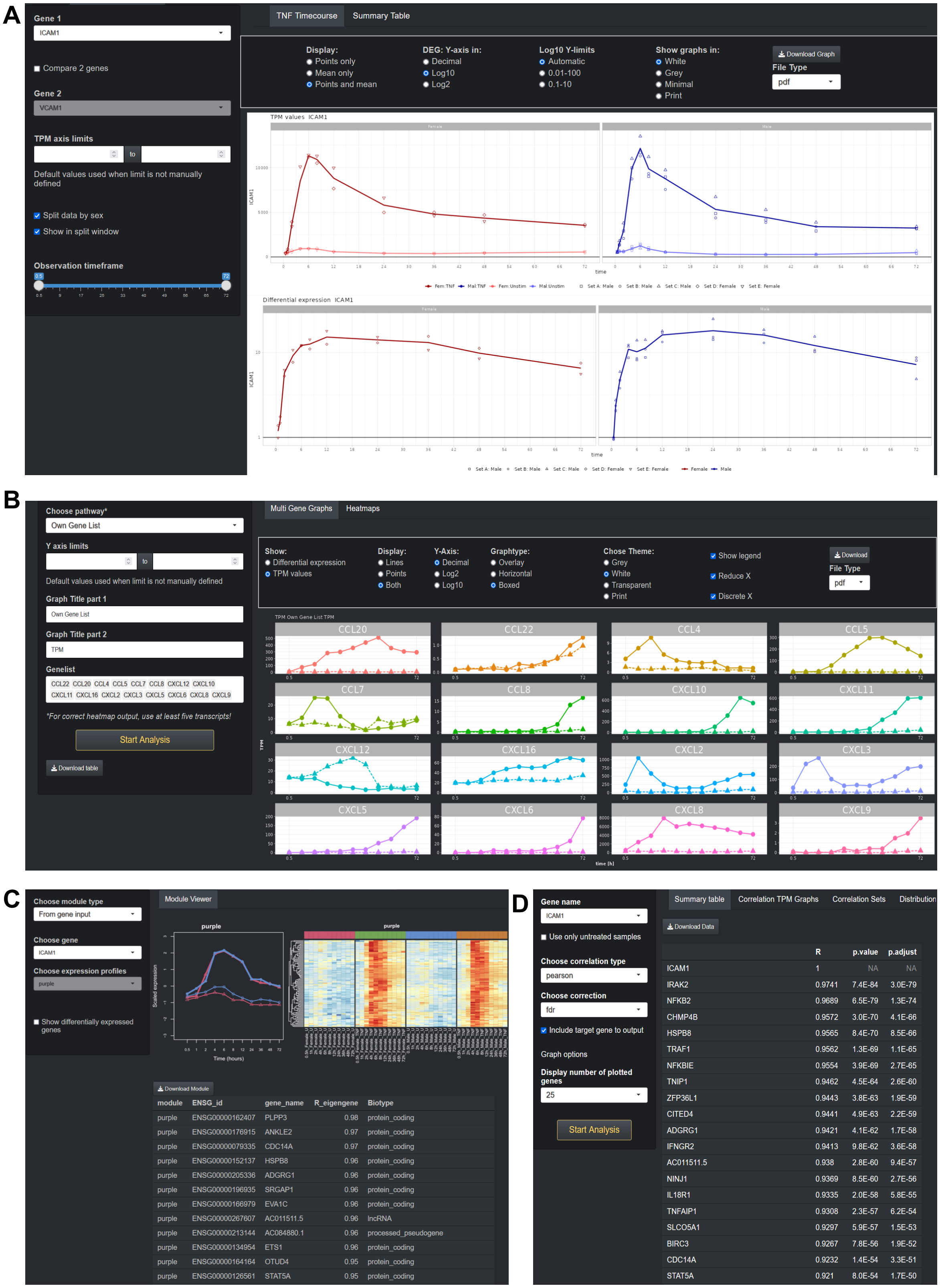

Figure S3. Website resource examples. All data generated in this study is available on http://www.endothelial-response.org/. Selected features include: (**A**) data viewer for temporal gene expression over time (displayed as absolute values and relative differential expression), (**B**) data plot generator to be used either with user defined gene lists, or predefined gene categories, selected from the dropdown menu (e.g., ´leukocyte recruitment´), (**C**) weighted network correlation analysis data section and module look up tool for any given input gene, (**D**) Expression similarity tool that can identify genes with the highest correlation to any given input gene across the dataset. Data plots can be downloaded as vector-images.
